# Communication Training Improves Sense of Performance Expectancy of Public Health Nurses Engaged in Long-Term Elderly Prevention Care Program

**DOI:** 10.5402/2012/430560

**Published:** 2012-11-19

**Authors:** Motoko Tanabe, Yoshimi Suzukamo, Ichiro Tsuji, Sin-Ichi Izumi

**Affiliations:** ^1^Department of Physical Medicine and Rehabilitation, Tohoku University Graduate School of Medicine, Sendai 980-8575, Japan; ^2^Department of Rehabilitation, Faculty of Health Science, Tohoku Fukushi University, Sendai 981-8522, Japan; ^3^Division of Epidemiology, Department of Public Health and Forensic Medicine, Tohoku University Graduate School of Medicine, Sendai 980-8575, Japan; ^4^Department of Rehabilitation Engineering, Graduate School of Biomedical Engineering, Tohoku University, Sendai 980-8575, Japan

## Abstract

This study examines the effectiveness of a communication skill training based on a coaching theory for public health nurses (PHNs) who are engaged in Japan's long-term care prevention program. The participants in this study included 112 PHNs and 266 service users who met with these PHNs in order to create a customized care plan within one month after the PHNs' training. The participants were divided into three groups: a supervised group in which the PHNs attended the 1-day training seminar and the follow-up supervision; a seminar group attended only the 1-day training seminar; a control group. The PHNs' sense of performance expectancy, and user's satisfaction, user's spontaneous behavior were evaluated at the baseline (T1), at one month (T2), and at three months (T3) after the PHNs' training. At T3, the PHNs performed a recalled evaluation (RE) of their communication skills before the training. The PHNs' sense of performance expectancy increased significantly over time in the supervised group and the control group (*F* = 11.28, *P* < 0.001; *F* = 4.03, *P* < 0.05, resp.). The difference score between T3-RE was significantly higher in the supervised group than the control group (*P* < 0.01). No significant differences in the users' outcomes were found.

## 1. Introduction

The service user's motivation is a fundamental element in the success of health promotion services for the elderly [1]. Service users who proactively participate in the service provider's health programs are better able to maintain or improve their health, and consequently, avoid the need for long-term care prevention program. Therefore, service providers should not only provide good health programs but also encourage service users to proactively participate in such programs. Increasing service users' participation in health promotion programs aimed at preventing the need for long-term care is important.

In Japan, a health care and rehabilitation service for preventing the need for long-term care, called “Long-term Care Prevention,” was established in 2005 [2, 3]. This service assesses elderly people living at home need to maintain or improve daily functions. The contents of this service are tailored to the user's health condition and include programs for exercise (e.g., stretching, balancing, and muscle building), improvement of oral function, and dietary improvement as well as health consultation and guidance for homebound service users and those with mental disorders such as dementia or depression. During the first year of the program, only 0.05% of the elderly aged 65 years and above participated, far below the estimated 5% [4, 5]. This low participation rate may be because the elderly did not feel the need for such services or their motivation to participate is low [6]. Therefore, measures to increase the motivation of users to participate in the long-term care prevention programs are needed. For the long-term care prevention service to succeed, it is important to define the users' daily needs and goals and to tailor the service accordingly [7].

The public health nurses (PHNs) who assess the participants' needs and who work with the participants to create an individualized long-term care prevention plan must be able to communicate well enough to help increase the users' motivation to participate. According to Wong et al., communication in the provider-patient relationship is an important determinant in patient satisfaction [8]. However, no method has yet been developed for motivating elderly people to participate in proactive health programs. Similarly, nor any method has yet been established for improving the communication abilities of PHNs. Consequently, PHNs are ill equipped to communicate with elderly individuals with sensitive or additional needs, particularly those who are depressed or who have lost hope. The PHNs' sense of performance expectancy [9] regarding their communication abilities needs to be enhanced through a training program that develops their communication abilities so that they can help motivate elderly individuals, particularly those with sensitive or additional needs.

In this study, we developed and implemented a communication skill training program that teaches PHNs to use coaching techniques to help service users achieve their goals. Recently, coaching has been introduced in the field of medicine and has been shown to be effective in areas such as self-management of patients with cardiovascular disease [10], pain control in cancer patients [11], pain assessment and management practices of pediatric nurses [12], negative attitude toward antidepressants of depressive patients [13], communication skills of pediatricians [14], support for patients with chronic diseases [15, 16], and the health behavior of the elderly [17].

This study aims to examine the effectiveness of a communication skill training based on a coaching theory for PHNs who were engaged in prevention care management. Specifically, we tested the following two hypotheses: (1) the training improves the PHNs' sense of performance expectancy regarding their communication skills; (2) the training increases the service users' satisfaction with the PHNs and their spontaneous behavior in their daily livings.

## 2. Methods

### 2.1. Participant Recruitment

In this study, the participants included 112 PHNs who belonged to comprehensive community support centers in City Y (located in central Japan and facing Tokyo Bay, and with a population of about 3,000,000), and the service users who met with these PHNs to create a long-term care prevention plan within one month after the PHNs' training.

The procedures of this study were implemented with approval from the institutional review board of the Tohoku University Graduate School of Medicine.

### 2.2. Training of PHNs

The communication skill training comprises two parts: a 1-day (seven hours) training seminarand a follow-up supervision. In developing the training program, we first interviewed the PHNs to ensure that the program's contents would be appropriate to the actual conditions of long-term care prevention management. The program deals with various communication skills, namely, “pacing,” “acknowledgement” “questions,” and “suggestions,” and includes lectures and role-playing exercises administered by master certified coaches accredited by the International Coach Federation.

The follow-up supervision was conducted for the PHNs who where selected at the attendance of the follow-up supervision. The PHNs were supervised 8 times during the three months after the 1-day training seminar. The coach used a telephone conference system and facilitated the group which made by 4-5 PHNs per group. The PHNs were trained for 30 minutes at once in this group coaching session. During this session, the PHNs and their coaches identified the problems and accomplishments of PHNs from applying their coaching skills to assist the service users. In addition, the PHNs participated in role-playing exercises supporting a conversation scene with a service user during this session.

### 2.3. Study Procedure

We divided the PHNs into three groups according to their selection of the schedule which they can participate in training and the attendance of follow-up supervision. These three groups are the supervised groups that participated in both the 1-day training seminar and the follow-up supervision; the seminar group that participated only in the 1-day seminar; the control group that did not participate in either the training or the follow-up supervision but received an explanation about the study's procedures and was given an outline of the coaching training. The PHNs recruited the service users to participate in the study—five users for the supervised group, four users for the seminar group, and three users for the control group.

The PHNs completed the questionnaire before the 1-day training seminar (T1) and one month (T2) and three months (T3) after the seminar. Meanwhile, the users completed the questionnaire at T2 and T3.

### 2.4. Assessment 

#### 2.4.1. Main Outcomes

The main outcome of the study was the PHNs' sense of performance expectancy regarding their communication abilities, measured by 12 items that were identified as behavioral characteristics of coach-type physicians in a web program titled “Coaching for Medical Interviews” (edited by Izumi et al.). The PHNs performed a self-evaluation at T1, T2, and T3. At T3, they also conducted a recalled evaluation (RE) of their communication skills before the training. We defined the difference score between T3 and RE as the PHNs' perceived improvement in their own communication skills owing to the communication training they received.

#### 2.4.2. Secondary Outcomes

As secondary outcomes, we evaluated the service users' “satisfaction with communication with the PHNs,” “general satisfaction with the PHNs,” and “self-rated spontaneous behavior in their daily livings.”

To measure satisfaction, we modified the American Board of Internal Medicine, Patient Satisfaction Questionnaire (ABIM-PSQ) [18] for the context of the long-term care prevention management. This scale comprises 11 items (total score of 0 to 55 points) regarding the users' satisfaction with their communication with the PHNs and 4 items (total score of 0 to 20 points) regarding the users' general satisfaction with the PHNs. To evaluate the users' spontaneous behavior in their daily livings, we used four items that measured “setting goals” and “action for goal achievement” based on the Coaching Skill Evaluation System that was adapted with permission from the Japan Coach Association [19].

In addition, we evaluated the PHNs' satisfaction with the 1-day training seminar through a 5-item questionnaire that was administered immediately after the seminar. Each item was evaluated from 1 (*strongly disagree*) to 5 (*strongly agree*).

#### 2.4.3. Moderator Variables

The moderator variables were the PHNs' characteristics (sex, age, years of experience in long-term care prevention management, and license as a PHN) and the user's characteristics (sex, age, support level (need support for daily activities) in Japan's long-term care prevention system, and Barthel Index of Activities of Daily Living (Barthel ADL Index)). 

### 2.5. Statistical Analysis

We compared the characteristics of the PHNs and users among the three groups. We used a one-way analysis of variance (ANOVA) and Tukey's honestly significant difference (HSD) test for the parametric variables and a chi-square test for the nonparametric variables.

Furthermore, we checked the factorial validity and reliability of the new scales used in this study (i.e., the PHNs' “sense of performance expectancy” and the users' spontaneous behavior in their daily livings). We conducted a principal factor analysis with a varimax rotation for validity and calculated the Cronbach's  *α*  coefficient for reliability.

To evaluate the effect of the training on the PHNs' communication skills, we first compared the PHNs' sense of performance expectancy scores among the three groups at T1, T2, and T3 with repeated measures by ANOVA. Next, we calculated the mean value and standard deviation during all the survey times and the difference scores among T1, T2, T3, and RE, for the three groups, which we then tested by a one-way ANOVA and Tukey's HSD test. To assess the PHNs' satisfaction with the 1-day training seminar, we calculated the ratio of PHNs who answered “strongly agree” in 5-item questionnaire. To assess the user's outcomes, we used a one-way ANOVA and Tukey's HSD test to determine the differences in the means among the three groups at T2 and T3. The analyses were performed using SPSS 16.0 (SPSS, Inc., Chicago, IL).

## 3. Results

### 3.1. Characteristics of Participants

**Figure 1:**
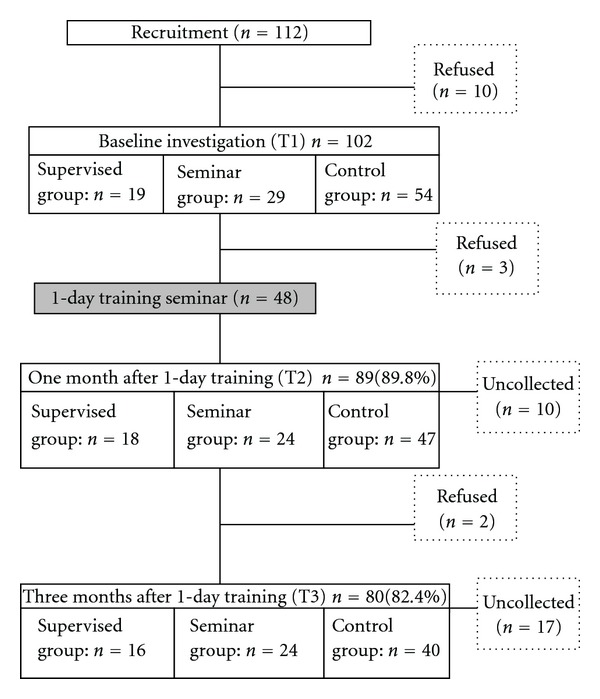
Flow chart of participants (public health nurses) during this study.

In this study, 102 out of the 112 PHNs recruited (91.1%) agreed to participate. Later, however, five PHNs dropped out for reasons related to retirement, jobs, or family. The remaining 97 PHNs were divided into three groups: 18 in the supervised group, 27 in the seminar group, and 52 in the control group. We were able to collect data from 80 out of 97 PHNs (82.4%) (Figure 1). In this study, 266 service users participated in this study and were divided into three groups: 84 in the supervised group, 85 in the seminar group, and 97 in the control group. There were no differences in terms of characteristics among the three groups of PHNs and service users (Table 1).

### 3.2. Factorial Validity and Reliability of the New Scales

The factor analysis of the 12 items measuring the PHNs' sense of performance expectancy revealed that the contribution ratio of the first factor was 44.8% and that the factor loadings of all items exceeded 0.5. This shows that the 12 items had a high unidimensionality, and hence, we then converted them into total scores. The Cronbach's  *α*  for these 12 items was 0.89.

Meanwhile, the factor analysis of the four items measuring the users' spontaneous behavior in their daily livings revealed that the contribution ratio of the first factor was 75.6%, and that the factor loadings of all the items exceeded 0.8. This shows that the four items had a high unidimensionality, and hence, we converted them into total scores. The Cronbach's  *α*  for these four items was 0.89.

### 3.3. Public Health Nurses' Sense of Performance Expectancy Regarding Their Communication Skills

There was a significant temporal increase in the scores of the PHNs' sense of performance expectancy for the supervised group (*P* < 0.001) and the control group (*P* < 0.05). However, there was no significant difference in the scores among the three groups at T1, T2, T3, and RE (Table 2). Regarding the difference scores among the various survey times, the difference score between T3 and RE was larger for the supervised group than for the control group (4.7 ± 4.1 versus 1.6 ± 3.3, *P* = 0.01).

We obtained the data for the 52 PHNs' satisfaction with the 1-day training seminar (Table 3). In reply to “Do you want to utilize the coaching skills in the support aspect of your long-term care prevention management role?” 67.8% of the PHNs answered “strongly agree.” When asked “Do you think the coaching skills will be useful in your interviews with prevention care users?” 61.5% of PHNs answered “strongly agree.”

### 3.4. Service Users' Satisfaction and Spontaneous Behavior

 There was no significant difference among the three groups regarding the service users' satisfaction with their communication with the PHNs, their general satisfaction with the PHNs, and their spontaneous behavior in their daily livings (Table 4). Similarly, no difference was observed even after adjusting for the service users' characteristics, namely, sex, age, support level, and Barthel ADL Index score.

## 4. Discussions

This is the first study to investigate the effectiveness of a training program that focuses on improving the communication skills of PHNs who play a pivotal role in Japan's elderly prevention care system. The results of the study indicated that the PHNs' sense of performance expectancy regarding their communication abilities improved after participating in both the training seminar and the follow-up supervision.

### 4.1. The Effect of Training on the Public Health Nurses' Communication Skills

In the supervised group, the improvement in the PHNs' sense of performance expectancy scores and the larger difference scores between T3 and RE showed that the PHNs felt that their skills have improved compared to those before the training. This indicates that the communication training that included the 1-day training seminar and the follow-up supervision may have improved the PHNs' sense of performance expectancy regarding their communication skills. A previous study showed that communication skills training significantly improved clinicians' and nurses' self-efficacy regarding skill implementation [20]. Likewise, the PHNs' perceived skill improvement in this study may be due to the fact that their self-efficacy regarding skill implementation had improved. Regarding the PHNs' satisfaction with the training, a significant percentage of the PHNs expressed interest in utilizing the coaching skills they learned in their prevention care management roles and agreed that the skills they learned would be useful. Moreover, these results suggest that the training improved the PHNs' sense of performance expectancy during their interviews with users in long-term care prevention management.

In their study, Arranz et al. found that counseling training for nurses improved the relationships between patients and their families [21]. Similarly, Yamagishi et al. found that assertion training for nurses improved the nurses' knowledge and usage of assertion, consequently reducing their work-related stress and mental workload [22]. Although we did not examine the PHNs' work-related stress in this study, it is possible that the improvement in the PHNs' sense of performance expectancy may improve their relationships with users and consequently reduce work-related stress.

The PHNs' sense of performance expectancy scores in the seminar group did not increase significantly compared to those in the supervised group. This indicates that the 1-day training may not be adequate for improving the PHNs' sense of performance expectancy.

Contrary to our expectations, the control group that did not receive training also show an improved sense of performance expectancy scores over time. A probable reason of this result is that the mere participation in this study raised awareness about the importance of effective communication with patients (i.e., the Hawthorne effect). However, the small difference scores between T3 and RE in the control group indicates that the PHNs' perception of their communication skills did not change significantly. This is in contrast to the significant increase in the supervised group's perception of their communication skills, indicating a significantly improved sense of performance expectancy.

### 4.2. Satisfaction and Spontaneous Behavior of Service Users

There was no significant difference among the three groups regarding the service users' satisfaction and spontaneous behavior in their daily livings at each survey time. There are two possible reasons for this result, which we discuss below.

First, the communication training may not have been sufficient for the PHNs to acquire all the skills necessary to improve user satisfaction and spontaneous behavior. Compared to a previous study on nurse-patient communication [23] that used 2-day training, our study used only 1-day training. Furthermore, our study may have been different from the previous study in terms of the PHNs' skill implementation; that is, whether the PHNs actually used the new skills in their interviews with the service users. Specifically, probably the service users who participated in this study may have already had good relationships with their PHNs, and thus, no significant improvement in user satisfaction and spontaneous behavior was observed. 

Second, probably that user satisfaction was not the proper measure. Specifically, it is possible that the user's satisfaction was influenced by the service provided rather than by the interviews with PHNs. The users were interviewed by the PHNs about once a month, whereas the services were provided continuously every week. Therefore, the interviews with the service provider staff were more frequent than that with the PHNs. Although the question items were about satisfaction with the PHNs' interviews, it may be that the users had a stronger impression of the services than of the interviews. It is possible then that this strong impression of the services influenced the evaluations of the interviews with the PHNs, as reflected in the results. The training aimed to improve the PHNs communication skills to encourage users to proactively participate in the long-term care prevention programs. Instead, of user satisfaction, the rate of attendance at and the contents of the services provided through the care plan may have been better outcome measures. For example, the level of goal attainment in the care plan may have been a better measure for the improvement in the patients' physical function or oral function.

Previous studies that dealt with coaching training used other outcome measures. A previous study on physical therapists who coached early rheumatism patients revealed that although the health perception and physical strength of patients in the training group improved, the patients' self-reported, physical activity did not change [24]. Another study that aimed to improve the self-efficacy of health care staff by training them on telephone coaching revealed that the physical activity and walking ability of patients improved significantly [25]. In sum, the results of these previous studies indicate that although coaching training of the health care staff improved the patients' physical functions, it did not improve the patients' self-reported outcomes. Thus, even though changes in the patients might have been observed if objective evaluation indices had been used, from the viewpoint of the users, the prevention care did not improve their physical functions.

The present study was limited in that we used the PHNs' self-evaluations of their sense of performance expectancy. Furthermore, we did not perform objective evaluations of the PHNs' communication skills before and after the training. In previous studies, the implementation of communication skills were monitored and recorded by a video system [26–28]. The use of this kind of objective evaluation of the PHNs' communication skills while considering the privacy of the patients may be worth considering in future studies.

## 5. Conclusions

In this study, the communication training based on a coaching theory improved the PHNs' sense of performance expectancy regarding their communication skills in their long-term care prevention management roles. The results of our study suggest that in order to determine the effects of the PHN training on the service users, it will be necessary to measure not only the users' subjective satisfaction but also objective outcome measures. In future studies, it may be necessary to expand the communication training to medical staff responsible for educating patients regarding the available health promotion and rehabilitation services so that such staff will be better equipped with the skills to motivate patients to proactively participate in such programs and consequently, improve the patients' physical functions. 

Communication skill training improves the sense of performance expectancy of PHNs engaged in prevention care and consequently, improves the care management of prevention care users. Such educational opportunities for PHNs should be increased.

## Figures and Tables

**Table 1:** The characteristics of participants.

	Supervised group *n* = 16	Seminar group *n* = 24	Control group *n* = 40	ANOVA* or chi-square test *P* value
Public health nurse				
Age, mean (SD)	46.1 (9.0)	45.0 (10.4)	41.1 (8.7)	0.123
Gender				
Female, *n* (%)	15 (93.8)	22 (91.7)	38 (95.0)	0.529
Male, *n* (%)	1 (6.2)	0 (0.0)	2 (5.0)	
Unknown, *n* (%)	0 (0.0)	2 (8.3)	0 (0.0)	
Licenses, *n* (%)				
Public health nurse	4 (25.0)	5 (22.7)	6 (15.0)	0.614
Nurse	14 (87.5)	20 (90.9)	38 (95.0)	0.610
Care manager	7 (43.8)	10 (45.5)	13 (32.5)	0.537
Supervisor of care manager	0 (0.0)	0 (0.0)	1 (2.5)	0.618

	*n* = 84	*n* = 85	*n* = 97	

Service users				
Age, mean (SD)	76.5 (8.3)	77.9 (6.5)	78.3 (6.4)	0.231
Gender (female), *n* (%)	55 (66.5)	58 (68.2)	76 (78.4)	0.128
Category of certification, *n* (%)				0.683
Specific elderly individuals	30 (35.7)	32 (37.6)	29 (29.9)	
Support level 1	14 (16.7)	15 (17.6)	17 (17.5)	
Support level 2	34 (40.5)	35 (41.2)	50 (51.5)	
Unknown	6 (7.1)	3 (3.5)	1 (1.0)	
BI^†^, mean (SD)	97.6 (5.3)	98.0 (4.9)	97.9 (5.6)	0.993
Number of comorbidity, mean (SD)	3.0 (1.8)	3.3 (2.0)	3.6 (1.9)	0.107
Live alone, *n* (%)	25 (29.8)	32 (37.6)	46 (47.4)	0.051
Depression diagnosis, *n* (%)	5	5	4	0.780

^∗^ANOVA: analysis of variance.

^†^BI: Barthel ADL Index score.

**Table 2:** Comparison of the sense of performance expectancy for public health nurse.

	Supervised group *n* = 16	Seminar group *n* = 24	Control group *n* = 40	ANOVA^‡^ *P* value
T1*, mean (SD)	32.6 (3.7)	35.1 (4.9)	32.9 (6.3)	0.229
T2*, mean (SD)	34.3 (3.1)	35.9 (6.1)	35.0 (6.6)	0.690
T3*, mean (SD)	37.1 (3.7)	36.5 (5.7)	35.6 (6.6)	0.630
RE^†^, mean (SD)	32.4 (3.6)	34.5 (6.2)	33.8 (6.9)	0.585
T2-T1, mean (SD)	1.7 (3.6)	0.8 (4.5)	2.3 (7.2)	0.651
T3-T1, mean (SD)	4.4 (4.4)	1.5 (4.1)	2.7 (7.2)	0.342
T3-T2, mean (SD)	2.8 (3.5)	0.4 (3.5)	0.5 (5.3)	0.194
T3-RE, mean (SD)	4.7 (4.1)	2.1 (2.7)	1.6 (3.3)	0.010

T1-T2-T3 main effects^§^ (*F*, *P*)	11.28, <0.001	1.15, 0.328	4.03, 0.022	

*T1: baseline assessment, T2: one month after 1-day training seminar, T3: three months after 1-day training seminar.

^†^RE: the score recalled evaluation of T1 on time T3.

^‡^ANOVA: analysis of variance.

^§^Results of repeated measures ANOVA.

**Table 3:** The public health nurses' satisfaction with the 1-day training seminar*.

	Strongly agree	Agree	Not sure	Disagree	Strongly disagree
Do you want to utilize the coaching skills in the support process of prevention care management?	35	15	2	0	0
	67.8%	28.8%	3.8%	0%	0%

Do you think the coaching skills will be useful in interviews with prevention care users?	32	19	1	0	0
	61.5%	36.5%	1.9%	0%	0%

Do you think that this training will be useful in the support of an in-home service provider and a designated in-home long-term care support provider?	22	21	8	1	0
	42.3%	40.0%	15.4%	1.9%	0%

Do you think that the contents of this training were easy to understand?	36	16	0	0	0
	69.2%	30.8%	0%	0%	0%

Do you think that the time distributions for lecture and role-playing practice in this training were appropriate?	22	23	6	0	0
	43.1%	45.1%	11.8%	0%	0%

*n* = 52 (supervised group or seminar group: *n* = 49, the other public health nurse: *n* = 3).

*Just after the completion of the 1-day training seminar.

**Table 4:** Comparison of service user's satisfaction and spontaneous behavior in one and three months after public health nurses' 1-day training seminar.

	Supervised group *n* = 84	Seminar group *n* = 85	Control group *n* = 97	ANOVA *P* value
Outcome of service user (T2)*				
Satisfaction with communication, mean (SD)	36.1 (6.0)	36.5 (6.4)	36.0 (6.1)	0.869
General satisfaction with public health nurse, mean (SD)	13.1 (2.4)	13.4 (2.3)	13.1 (2.2)	0.556
Spontaneous behavior, mean (SD)	11.2 (2.7)	11.3 (2.8)	11.1 (2.9)	0.898

Outcome of service user (T3)^†^				
Satisfaction with communication, mean (SD)	35.2 (6.2)	35.6 (6.1)	35.6 (5.7)	0.872
General satisfaction with public health nurse, mean (SD)	12.9 (2.4)	13.0 (2.3)	11.2 (2.6)	0.914
Spontaneous behavior, mean (SD)	10.9 (3.1)	10.9 (3.0)	11.2 (2.7)	0.763

*T2: one month after the 1-day training seminar.

^†^T3: three months after the 1-day training seminar.
